# Oxytocin receptor single nucleotide polymorphism predicts atony-related postpartum hemorrhage

**DOI:** 10.1186/s12884-022-05205-w

**Published:** 2022-11-29

**Authors:** Elise N. Erickson, Kathleen M. Krol, Allison M. Perkeybile, Jessica J. Connelly, Leslie Myatt

**Affiliations:** 1 Present Address: University of Arizona, Tucson, AZ USA; 2 Oregon Health and Science University, OR Portland, USA; 3 University of Virginia, Charlottesville, VA USA

**Keywords:** Oxytocin receptor, Pharmacogenomics, Genetics, Postpartum hemorrhage, Third stage labor

## Abstract

**Background:**

Postpartum hemorrhage remains a key contributor to overall maternal morbidity in the United States. Current clinical assessment methods used to predict postpartum hemorrhage are unable to prospectively identify about 40% of hemorrhage cases. Oxytocin is a first-line pharmaceutical for preventing and treating postpartum hemorrhage, which acts through oxytocin receptors on uterine myocytes. Existing research indicates that oxytocin function is subject to variation, influenced in part by differences in the DNA sequence within the oxytocin receptor gene. One variant, rs53576, has been shown to be associated with variable responses to exogenous oxytocin when administered during psychological research studies. How this variant may influence myometrial oxytocin response in the setting of third stage labor has not been studied. We tested for differences in the frequency of the oxytocin receptor genotype at rs53576 in relationship to the severity of blood loss among a sample of individuals who experienced vaginal birth.

**Methods:**

A case–control prospective design was used to enroll 119 postpartum participants who underwent vaginal birth who were at least 37 weeks of gestation. Cases were defined by either a 1000 mL or greater blood loss or instances of heavier bleeding where parturients were given additional uterotonic treatment due to uterine atony. Controls were matched to cases on primiparity and labor induction status. Genotype was measured from a maternal blood sample obtained during the 2^nd^ postpartum month from 95 participants. Statistical analysis included bivariate tests and generalized linear and Poisson regression modeling.

**Results:**

The distribution of the genotype across the sample of 95 participants was 40% GG (*n* = 38), 50.5% AG (*n* = 48) and 9.5% AA (*n* = 9). Blood loss of 1000 mL or greater occurred at a rate of 7.9% for GG, 12.5% for AG and 55.6% for AA participants (*p* = 0.005). Multivariable models demonstrated A-carriers (versus GG) had 275.2 mL higher blood loss (95% CI 96.9–453.4, *p* < 0.01) controlling for parity, intrapartum oxytocin, self-reported ancestry, active management of third stage or genital tract lacerations. Furthermore, A-carrier individuals had a 79% higher risk for needing at least one second-line treatment (RR = 1.79, 95% CI = 1.08–2.95) controlling for covariates. Interaction models revealed that A-carriers who required no oxytocin for labor stimulation experienced 371.4 mL greater blood loss (95% CI 196.6–546.2 mL).

**Conclusions:**

We provide evidence of a risk allele in the oxytocin receptor gene that may be involved in the development of postpartum hemorrhage among participants undergoing vaginal birth, particularly among those with fewer risk factors. The findings, if reproducible, could be useful in studying pharmacogenomic strategies for predicting, preventing or treating postpartum hemorrhage.

**Supplementary Information:**

The online version contains supplementary material available at 10.1186/s12884-022-05205-w.

## Background

Postpartum hemorrhage (PPH) remains a primary contributor to global maternal morbidity and mortality [1]. PPH is a clinical diagnosis based on any accumulated blood loss causing signs/symptoms of hypovolemia after birth and/or at least 1000 mL cumulative blood loss in 24 h after birth [2]. The etiology of PPH is multifactorial. Uterine atony [3], morbidly adherent placentation [4], genital tract trauma [5] and rare underlying coagulopathies [6] may each directly contribute to the accumulated blood loss, though atony is considered to be a main contributor. Importantly, uterine atony-linked PPH increased 14% among induced labors in the United States (US) followed by vaginal birth and increased 61.1% for all Cesarean births between 2002–2012 [7]. PPH can be life threatening, requiring multiple pharmaceutical therapies or additional interventions (uterine compression/ tamponade, embolization or hysterectomy), blood transfusion or prolong hospitalization [8, 9]. Experiencing a PPH can interfere with maternal/infant bonding, or lead to traumatic stress or anemia [10, 11]. Suboptimal lactation [12] and postpartum mood disorders [13–15] are also associated with PPH. Furthermore, more recent analysis using US data noted that of those who had PPH, 20–25% experienced subsequent severe maternal morbidity, and also noted disparities by racial/ethnic identity [16].

Pharmaceutical oxytocin is routinely recommended and administered after birth to help prevent PPH by stimulating sustained uterine contraction after birth of the newborn [17]. However, while prophylactic oxytocin (active management of the third stage of labor) has been recommended for at least 20 years [1], PPH continues to occur. Furthermore, rates of blood transfusions or interventions to control PPH in the US doubled from 2002 to 2012 (across all vaginal and Cesarean births), indicating that PPH remains a persistent problem [7].

A key component of clinical preparedness and response to PPH is the use of a clinical risk assessment tool [18]. These tools help obstetric providers and nurses systematically monitor for associated risk factors and prepare for possibility of PPH and for earlier identification of the problem. However, clinical assessment tools that are used to predict PPH before the birth, do not predict about 40% of PPH cases [8, 19]. That is, females giving birth are coded as “low-risk” according to the assumed risk factors embedded in the tool, yet later develop PPH anyway. Factors such as placenta previa or abruption trigger high risk PPH scores, yet occur less frequently in the overall population. It is therefore likely that a large percentage of the overall childbearing population will have a lower risk status, making early prediction of PPH among this group challenging as PPH occurs less frequently within the low-risk scoring group.

Epidemiologic evidence suggests PPH could be influenced by genetics; however, this has not been tested prospectively. For example, large population studies demonstrate that people who experience PPH may be likely to have a reoccurrence of PPH in a subsequent birth [20, 21]. In addition, a family history of PPH (maternal/paternal) is associated with higher PPH odds as well [22]. Finally, as mentioned, various demographic groups, which may have common ancestry are also associated with higher prevalence of PPH [16, 23, 24]. Individuals with Hispanic/Latina, Native American/ Indigenous and Asian ancestry have been identified as having elevated risks for PPH compared to European ancestral groups [24, 25]. A systematic review from 2008 noted broad global variability, with the highest rates of severe PPH (defined as 1000 mL) in Latin American countries at 5.3% of births, compared to 2.2% among African countries and 1.78% among Asian countries [26]. Some studies have documented that African American subgroups, have higher rates of PPH, but this is a less consistent finding, and one review highlighted that uterine atony related PPH was not more common among African American individuals [25]. Importantly, morbidity appears to be higher for African American individuals when PPH occurs [16], possibly related to disparities in appropriate/timely medical care—as implementation of a PPH specific standardized toolkit appears to reduce disparities in morbidity from PPH [27]. Despite advances in addressing PPH through better treatment, the likelihood of PPH occurring has not been studied robustly from a genetic perspective.

Oxytocin serves an important role during the final stages of labor and birth [28], in particular, binding to myometrial oxytocin receptor (OXTR), causing uterine contraction [29] and helping to expel the placenta and prompt uterine involution—thereby minimizing bleeding. Genetic differences within the oxytocin system could functionally modulate contractions, positing it as an intriguing candidate for consideration of risk factors for PPH. The *OXTR* gene is located within a 17 kb region on chromosome 3p25 [30] and translates into OXTR, a G-protein coupled receptor [31, 32]. Of many reported single nucleotide polymorphisms (SNPs) in the *OXTR*, rs53576 has been among the most widely studied [33]. Variant rs53576 is found in the third intron and varies by the bases adenine (*A*) or guanine (*G*) [34]. This SNP has been informative in examining variability in OXTR function, however these studies were primarily conducted in brain tissue specimens and/or reporting on the role of oxytocin/OXTR in psychological or behavioral contexts (e.g. eating disorders [35], substance use disorder [36], depression [37–39], social behavior [40]. Studies in psychology on the use of intranasal oxytocin administration on neural pathway outcomes that are oxytocin-dependent indicated that GG individuals had a more robust response to exogenous oxytocin. These studies included those examining the consolidation of intrusive memories among healthy women [41], amygdala responses to faces [42], measures of self-perception [43] and social cooperation [44] as well as in modulating symptoms of autism spectrum disorder [45]. According to the Genotype-Tissue Expression project (GTEx) [46] the A / G alleles are associated with differential splicing of the intron/exon of the transcript in breast tissue and with differential *OXTR* gene expression within several brain regions. GTEx data for 129 uterine samples show an expression quantitative trait locus (eQTL) m-value of 0.4 (*p* = 0.11), however, these samples are most likely not from pregnant donors, when OXTR is widely upregulated and available in uterine tissue. Together, this background informed our hypothesis that A-carriers may be less responsive to oxytocin during the birth process and would be more prone to having heavy bleeding or PPH occur after birth.

Therefore, in this study, we examined the *OXTR* allele frequency for the rs53576 for individuals with PPH/ heavy postpartum blood loss after vaginal birth that was specifically attributed to uterine atony or inadequate uterine tone. We compared outcomes for these cases of PPH to outcomes for matched control participants who had physiologic bleeding after birth.

## Methods

We used a case–control design for this prospective study. Postpartum hemorrhage cases were matched to participants with physiologic blood loss after birth based on induction of labor status (vs. spontaneous labor onset) and primiparity (vs. multiparity). Institutional ethics board at (Oregon Health and Science University) approved this protocol. Informed consent process occurred during a face-to-face encounter and written informed consent was obtained upon enrollment in the study. All study materials were translated into Spanish and telephonic interpreter services were used when needed.

## Participants

Individuals who gave birth in the greater Portland, Oregon region including hospital and community birth settings (home or birth center) were invited to participate. Participants giving birth in our medical center were approached in the postpartum unit on the first or second day postpartum. Participants in the broader community responded to advertisements posted on social media or to posted flyers. Enrollment criteria included the following: vaginal birth, 37 weeks of gestation or greater, English or Spanish-speaking, over 15 years of age. Further exclusions for enrollment as a case of PPH/heavy bleeding included having a known hereditary coagulopathy (e.g. vonWillebrand) or prothrombotic condition treated with anticoagulant therapy during pregnancy, hemolysis-elevated liver enzymes-low platelet (HELLP) syndrome, disseminated intravascular coagulation, magnesium sulfate administration during labor, tocolysis for preterm labor during the same admission episode, placenta accreta, vasa previa, gestational thrombocytopenia with less than 80,000 platelets/microliter or a severely bleeding genital laceration that was reportedly the source of the PPH. Participants completed baseline survey measures upon enrollment and at the blood sampling visit using REDCap research database [47].

Due to the inaccurate nature of blood loss estimation after birth [48–50] as well as practitioner variability in prevention/treatment of PPH [51–53], we identified cases of PPH using a robust description of the birth events. Cases were identified if the birth events met one of the following: cumulative blood loss >  = 1000 mL (either reported as estimated or measured blood loss) in the first two hours after birth *and* bleeding was attributed to lack of uterine tone or atony, or blood loss of at least 400 mL in the first two hours after birth with documentation of uterine atony *and* additional uterotonic medication (oxytocin, misoprostol, methylergonovine, and/ or carboprost tromethamine) administered at the time of birth to treat heavier than normal bleeding (per provider documentation), again not attributed to lacerations.

The rationale for having this broader definition was related to the understanding that blood loss is often underestimated by physicians and midwives and that preventive treatments or additional uterotonic medication for initial heavy bleeding would have most likely reduced subsequent blood loss thereby avoiding a PPH diagnosis based solely on the volume of blood loss.

Between 6–10 weeks after birth, maternal blood samples for DNA were obtained. The sampling was delayed until after the first month postpartum to limit the possibility of chimerism if a participant was given a blood transfusion following birth, and to limit invasive sampling given that half the participants just experienced a clinically significant blood loss. The sample was collected into an EDTA tube from a peripheral venipuncture by trained research assistants or phlebotomists and immediately stored in -80 °C freezer.

### Data acquisition / genotype

DNA isolation was performed using reagents and procedures outlined in the QIAamp DNA Mini Kit (Qiagen, Hilden, Germany). All DNA was quantitated using Nanodrop. Each DNA sample was amplified by polymerase chain reaction (PCR) for the rs53576 region target with the primers 5′-GCCCACCATGCTCTCCACATC-3′ and 5′- GCTGGACTCAGGAGGAATAGGGAC-3 using a 9700 GenAmp thermocycler and Amplitaq gold 360 PCR reagents following the manufacturer’s instructions, (ThermoFisher Scientific Waltham, MA 02,451). Following PCR samples were sequenced using a BigDye Terminator v3.1 cycle sequencing kit and the products analyzed on a 3130XL Genetic analyzer (ThermoFisher).

### Medical record abstraction and oxytocin administration calculation

Medical records were reviewed and clinical data was abstracted by study staff. We examined the total dose, duration and maximum dosage of oxytocin, as well as comorbidities, blood loss events and treatment including oxytocin and other uterotonic medications. Oxytocin start and stop times were recorded during labor, along with the timing and duration of titration of oxytocin doses. The dosing of oxytocin is quantified in milliunits (mU)/min, therefore the total cumulative dosage was a product of the duration (min) of a given level of titration and the oxytocin dose (e.g. 60 min × 2 mU/min = 120 mU). Prenatal, intrapartum or postpartum complications were also noted.

Similarly, oxytocin given after birth for active management of the third stage of labor was recorded as well as other treatments beyond prophylaxis for heavier bleeding including intravenous (IV) oxytocin, intramuscular oxytocin, misoprostol, methylergonovine, carboprost tromethamine, tranexamic acid, bimanual compression, uterine massage, bladder management, manual removal of retained clots or placental/membrane fragments. Depending on the institution (participants gave birth in 5 different institutions and 2 outside of a hospital setting), active management of third stage labor may consist of 10 U of oxytocin given via intramuscular injection or through the intravenous line. In some cases, nurses will instead utilize the existing intravenous solution of oxytocin being used for labor management, which may be 30 U oxytocin in 500 ml lactated ringers (however, some institutions may use 40 U in 1000 ml). Providers would typically also order additional IV solution of oxytocin, administered rapidly if bleeding was brisk following birth, regardless of prior prophylaxis. We noted the dose, duration and the indication for each postpartum medication. For some births, participants did not have any postpartum oxytocin administration, if the person declined the medication for active management of third stage and/or the practitioner assessed the patient to be low-risk for postpartum hemorrhage. Finally, we recorded intravenous iron therapy or blood transfusions as well as delayed postpartum hemorrhage (> 2 h after birth). In the enrollment survey, participants were asked to self-report their pregnancy, birth and medical history, which helped to validate medical record findings.

### Statistical methods

Differences in baseline characteristics, demographics and birth-related outcomes were compared by bivariate statistical tests (Spearman ρ, χ^2^, t-tests, Wilcoxon Rank tests as appropriate) between cases and controls. Hardy–Weinberg Equilibrium χ^2^ tests were conducted. All *p*-values are 2-tailed unless indicated with the level of significance set at < 0.05. Statistical analyses were performed with Stata SE 17 with figures generated in R. Allele frequencies were quantified and compared to published frequencies of 60% G/G, 30% A/G and 10% A/A for European/Latin American and African populations. Asian populations, however, have been reported to have a different distribution overall, with GG being less frequent [54]. We compared baseline, demographic and pregnancy characteristics across the sample between *A*-carriers and non-*A* carriers (AA/AG vs. G/G) using appropriate bivariate statistics, followed by a three-way comparison across the genotypes G/G, A/G, G/G with χ^2^ and Kruskall-Wallis tests as indicated.

We tested for allelic frequency differences between cases and controls as they were defined for the study enrollment. We then also compared allele frequency to 1) blood loss of greater than 400 mL and 2) blood loss of greater than or equal to 1000 mL and 3) median (IQR) blood loss as a continuous outcome variable. Using a generalized linear model (GLM) (with a gamma distribution due to the skewed nature of the blood loss outcome and an identity link) we estimated the quantity of total blood loss between A-carriers and G/G homozygous individuals. Covariates relevant to total blood loss and the genotype predictor included: European ancestry, oxytocin dosage during labor, primiparity, presence of genital tract lacerations (as these can be a source of bleeding) and prophylactic oxytocin via active management of the third stage of labor. Next, we conducted an interaction analysis with the total dosage of oxytocin used during labor with the A-carriers vs. G/G. We adjusted for self-reported ancestry in the second model. In the third model, we added primiparity and in a final fourth model we controlled for the presence of genital tract lacerations. Finally, a Poisson regression model was used to examine relative risk for requiring treatment for PPH using other pharmaceutical treatments/second-line medications (misoprostol, methylergonovine, carboprost tromethamine or tranexamic acid) using genotype as the primary predictor and adjusting for the same covariates as prior analyses (primiparity, ancestry, genital lacerations and active management of third stage labor).

## Results

### Sample

**Fig. 1:**
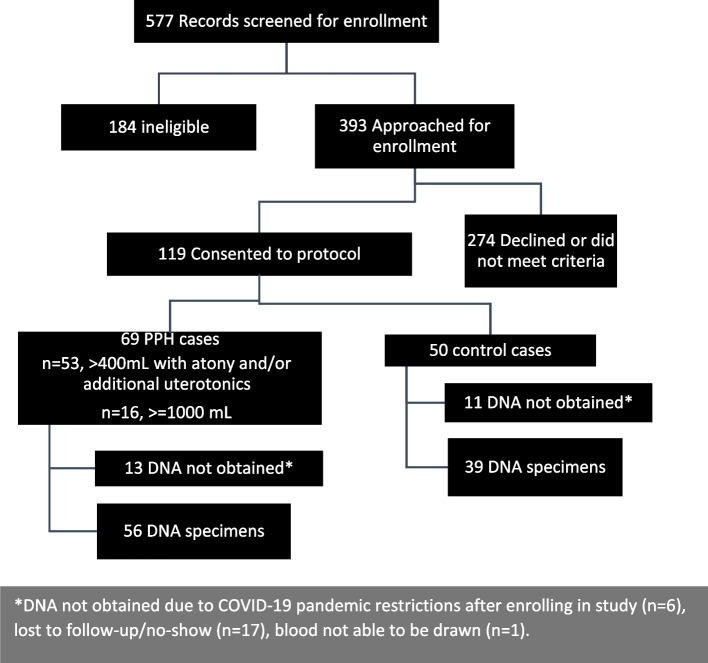
Study enrollment flow diagram: case–control study of postpartum hemorrhage among vaginal births

Screening and enrollment of participants took place between November 2018 through February 2020, study procedures including recruitment and study visits were put on hold due to COVID-19 pandemic restrictions, thereby limiting the intended sample size of 200 (Fig. 1).

A total of 119 participants enrolled in the study and 95 DNA samples were available for analysis. The intent for this study was a 1:1 match of cases to control participants. However, in the end, cases outnumbered controls (69 to 50). We therefore decided to present our results comparing cases to control participants in the supplement as intended and also using total blood loss as a continuous outcome with appropriate controls due to the sampling strategy and limitations.

The characteristics of the 119 enrolled in the study as either a case of PPH or a control participant are included in the Supplemental Table 1. Differences between case and control participants included body mass index, with PPH cases having significantly higher body mass index (BMI) at delivery (31.9 kg/m^2^ vs. 29.9 kg/m^2^, *p* = 0.02). Despite efforts to control for parity on enrollment, 57% of cases were primiparous compared to 38.0% of controls (*p* = 0.03). More participants enrolled as cases self-identified with Latin American ancestry or as Hispanic (15.9% cases / 2% control). While the number of participants who needed oxytocin in labor did not differ between cases/controls (cases were matched on labor induction status), the total quantity, duration and maximum dosage needed during labor (as well as for postpartum treatment) was higher among cases. First, second and third stage labor duration did not differ on average between cases/controls, nor did duration of ruptured membranes. Instrument assisted birth, prior cesarean birth and presence of any genital tract trauma were also similar between groups. By design, median (IQR) total blood loss for cases was higher at 600 mL (500–900 mL) compared to a median of 250 mL (150–350 mL) among controls.

**Table 1:** *OXTR* genotype (rs53576) and oxytocin administration and postpartum hemorrhage for 95 vaginal births

	*OXTR* G/G 38 (40.0%)	*OXTR* AG/AA 57 (60.0%)
**Demographic characteristics**		
Participant age (years), mean (SD)	33.1 (4.4)	33.9 (4.5)
Primiparous, n (%)	13 (34.2)	30.0 (52.6)
Self-reported ancestry *(may have 1 or more)*		
European, n (%)	35 (92.1)	44 (77.2)
Latin American, n (%)	2 (5.3)	6 (10.5)
Asian, n (%)	2 (5.3)	10 (17.5)
African, n (%)	1 (2.6)	0 (0.0)
Other, n (%)	3 (7.9)	4 (7.0)
Case, n (%)	19 (50.0)	20 (35.1)
Control, n (%)	19 (50.0)	37 (64.9)
**Antenatal characteristics**		
Body Mass Index at delivery (kg/m^2^), mean (SD)	30.2 (4.2)	31.6 (5.5)
Gestational diabetes, n (%)	6 (15.8)	9 (15.8)
Hypertension /preeclampsia (without severe features), n (%)	2 (2.3)	7 (12.3)
Fibroid uterus, n (%)	0 (0.0)	2 (3.5)
Anemia during pregnancy, n (%)	7 (18.9)	15 (26.3)
**Labor and Birth Characteristics**		
Spontaneous onset of labor, n (%)	20 (52.6)	27 (47.3)
Fever/ suspected intrapartum infection, n (%)	2 (5.3)	4 (7.0)
IV antibiotics during labor, n (%)	14 (36.8)	13 (22.8)
Gestational age at birth (weeks), mean (SD)	39.6 (1.3)	39.9 (1.2)
No oxytocin in labor, n (%) Oxytocin in labor, n (%)	20 (52.6) 18 (47.4)	20 (35.1) 37 (64.9)
Total oxytocin used in labor (Units), median (IQR)	5.3 (0.6–12.6)	3.7 (1.3–12.3)
Total oxytocin used postpartum (Units), median (IQR)	10 (10–23.6)	15.0 (10.0–25.0)
Maximum dose oxytocin used in labor (mU/min), median (IQR)	9.5 (6.0–17.0)	12 (6.0–17.0)
Duration oxytocin used in labor (hours), median (IQR)	13.0 (3.9–20.9)	11.4 (6.9–19.0)
Duration first stage labor (hours), median (IQR)	10.5 (5.5–19.7)	11.6 (7.7–18.3)
Duration second stage labor (hours), median (IQR)	0.8 (0.3–1.8)	1.0 (0.4–1.9)
Length of third stage of labor (minutes), mean (SD)	11.1 (12.5)	9.0 (9.1)
Female infant sex, n (%)	21 (55.3)	27 (47.4)
Newborn weight (grams), mean (SD)	3396.4 (697.4)	3481.3 (637.0)
**Postpartum Characteristics**		
Postpartum blood loss > = 400 mL, n (%)	18 (47.4)	43 (75.4)**
Postpartum blood loss > = 1000 mL, n (%)	3 (7.8)	11 (19.3)
Total blood loss (mL), median (IQR)	400 (200–600)	500 (423–800)**
Retained placenta/fragments, n (%)	5 (13.2)	5 (8.8)
Medications used for bleeding/hemorrhage, n (%)		
Active management of third stage labor (Oxytocin IM/IV for prevention)	34 (89.5)	47 (82.5)
Active management of third stage labor (Oxytocin IM/IV for prevention)	15 (42.9)	25 (43.8)
Received one or more second-line pharmaceutical treatment (misoprostol, methylergonovine, carboprost tromethamine or tranexamic acid)	12 (31.8)	33 (57.9)*
Other hemorrhage interventions, n (%)		
Bimanual compression	3 (7.8)	13 (22.8)*
Dilatation and curettage	1 (2.6)	3 (5.3)
Bakri Balloon	0 (0.0)	2 (3.5)
Difference in hemoglobin (third trimester to lowest postpartum value in mg/dL), mean (SD)	-0.6 (1.3)	-1.6 (2.2)*
Received iron infusion or blood transfusion, n (%)	3 (7.8)	9 (15.8)
No genital trauma (none/no repair needed), n (%)	14 (40.0)	13 (22.8)

^*^p < 0.05, **p < 0.01, ***p < 0.001

The distribution of the rs53576 alleles across the sample of 95 participants with DNA was 38 (40.0%) G/G, 48 (50.5%) A/G and 9 (9.5%) A/A. This distribution follows similar and expected frequencies reported by Butovskaya et al. (2016) among the 1000 Genomes project for European, Latin American, and African populations. The distribution in Asian populations for rs53576 has been reported to have a significantly higher percentage of A-carriers (G/G = 10.5%, A/G = 42.3%, A/A = 47.2%). Among G/G participants, 50% were enrolled as PPH cases and 50% as control participants. Among A-carrier participants 37 (64.9%) were cases and 35% were controls (*p* = 0.15). No other baseline differences were noted to be significantly different by the genotype of the participants (Table 1). The Hardy–Weinberg tests for both cases and controls were non-significant (case χ^2^(1) = 1.62, *p* = 0.20, control χ^2^(1) = 0.09, *p* = 0.76).

### A-carriers experienced greater postpartum bleeding

**Fig. 2:**
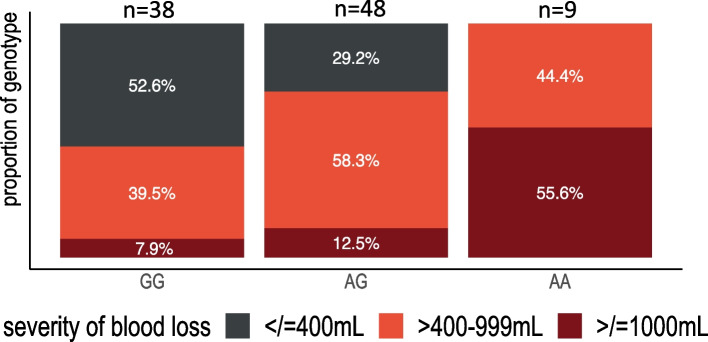
Severity of blood loss after birth across the rs53576 *OXTR* genotype. Fisher’s exact χ^2^(4) = 19.9, *p* = 0.001, frequency of severity of blood loss between genotypes

Despite having many similarities among pregnancy characteristics, A-carrier participants had a higher median total blood loss after giving birth (*p* = 0.009) (Fig. 2). Among A-carriers, 75.4% (*n* = 43) had a blood loss estimated at 400 mL or higher (*p* = 0.005), compared to 47.4% (*n* = 18) for G/G individuals. Further, only 3 G/G participants (7.8%) experienced a hemorrhage at using the definition of 1000 mL or greater compared to 19.3% of A/G and A/A participants (*p* = 0.13). We tested this difference across all three genotypes and found that A/A participants experienced the highest blood loss, with all but 2 individuals experiencing 500 mL or greater blood loss (the other two were reported as 450 mL and 480 mL). Overall A/A homozygotes were more likely to have a PPH by the 1000 mL definition (55.6%) compared to the individuals carrying AG (12.5%) or GG (7.9%) alleles (Fisher’s exact χ2 (2), *p* = 0.005).

### Genotype predicts uterotonic needs postpartum, but not for labor stimulation

Genotype did not vary between individuals who did or did not need oxytocin for labor stimulation. Oxytocin was used in 64.9% of A/G and A/A participants’ labors and in 47.4% of G/G participants (*p* = 0.09). For those needing oxytocin in labor, the median (IQR) total dosage was 3.7 total Units (1.3–12.3) for A/G or A/A individuals and 5.3 (0.6–12.6) for GG *(p* = 0.68). Similarly, neither peak dose (maximal mU/min) nor duration of infusion differed between the genotype groups. Further, no differences were seen in the median length of labor (first, second, or third stages). However, second line medication use for controlling bleeding was more commonly needed for A-carriers, with 57.8% received treatment for heavy bleeding using 1 or more second line pharmaceuticals, whereas only 31.6% of G/G individuals received second-line medications (Fisher’s exact, *p* = 0.002).

Using generalized linear regression models, we further tested the associations between genotype and blood loss (Table 2). For each of the 5 models we found that A-carrier individuals had higher volumes of bleeding. This remained significant while controlling for the potential role of ancestry on genotype, and the associations of oxytocin use in labor, parity, genital tract lacerations and prophylactic oxytocin administration (active management of third stage labor) with total blood loss after birth. Across the models, the total blood loss associated to genotype was between 200 and 300 mL higher for A-carrier individuals compared to G/G. In addition to genotype, higher oxytocin dosage needed during labor was also consistently associated with higher blood loss in Models 2–5 (Table 2).

**Table 2:** Generalized linear model of blood loss after vaginal birth by *OXTR* genotype (rs53576) accounting for relevant covariates

	**Total blood loss volume** β(95%CI)
**Model 1**	
*OXTR* A/G & A/A (vs. GG)	203.78 (6.01 – 401.54)*
European Ancestry vs. None	40.77 (-219.51 – 301.07)
**Model 2**	
* OXTR* A/G & A/A (vs. GG)	300.76 (126.61 – 474.92)***
European Ancestry vs. None	110.21 (-112.84 – 333.27)
Oxytocin dosage during labor (per Unit)	24.20 (7.64 – 40.76)**
**Model 3**	
* OXTR* A/G & A/A (vs. GG)	275.24 (96.33 – 454.16)**
European Ancestry vs. None	147.18 (-79.13 – 373.49)
Oxytocin dosage during labor (per Unit)	20.85 (3.64 – 38.07)*
Primiparity	125.77 (-68.33 – 319.88)
**Model 4**	
* OXTR* A/G & A/A (vs. GG)	278.09 (100.36 – 455.83)**
European Ancestry vs. None	133.36 (-94.05 – 360.77)
Oxytocin dosage during labor (per Unit)	20.68 (-3.08 – 38.28)*
Primiparity	95.89 (-124.97 – 316.76)
Genital tract lacerations	29.46 (-55.09 – 127.26)
**Model 5**	
* OXTR* A/G & A/A (vs. GG)	275.16 (96.92 – 453.40)**
European Ancestry vs. None	148.99 (-75.19 – 373.18)
Oxytocin dosage during labor (per Unit)	21.25 (3.74 – 38.75)*
Primiparity	21.25 (3.74 – 38.75)*
Active Management of Third Stage Labor	-33.04 (-259.81 – 193.72)

^*^models including Latin American ancestry as a covariate were not materially different than controlling for only European ancestry

### Quantity of oxytocin used during labor and genotype interact to predict postpartum hemorrhage

**Fig. 3:**
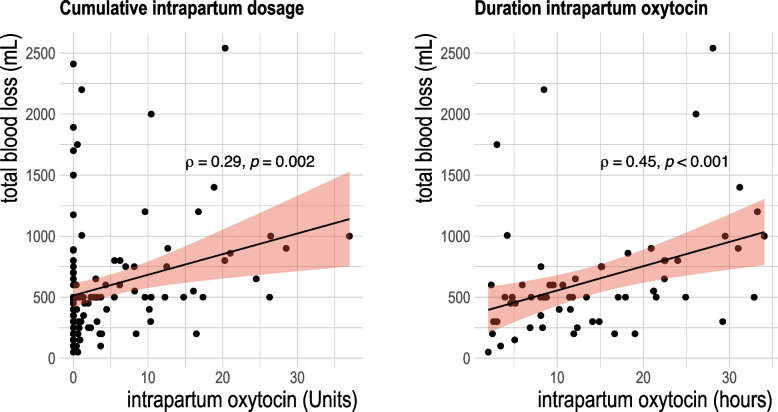
Intrapartum oxytocin use correlated with total blood loss after birth abstracted from the electronic health record. Left denotes cumulative oxytocin administered throughout labor, Right denotes total duration of oxytocin infusion during labor

**Fig. 4:**
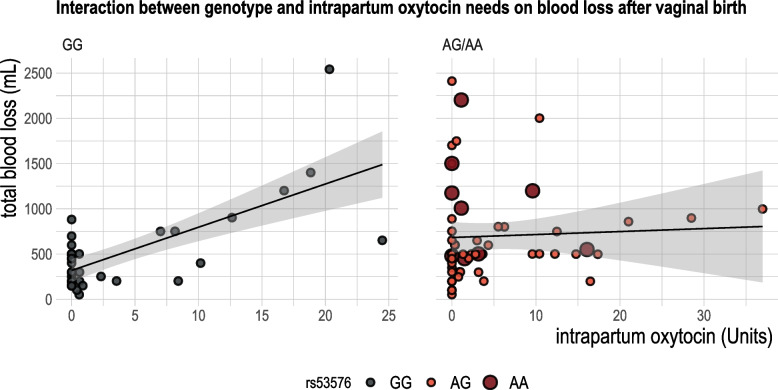
Interaction between genotype and intrapartum oxytocin needs on blood loss after vaginal birth. Interaction statistics listed in Table 3. Left denotes GG participants and increased positive correlation between greater oxytocin administration and higher blood loss. Right denotes AG/AA participants (larger red circles = AA) and a diminished effect of greater exposure to intrapartum oxytocin on eventual total blood loss

Bivariate statistics showed that both the cumulative dosage and duration of oxytocin used in labor were significantly associated with greater blood loss (Fig. 3). We then examined an interaction between genotype and oxytocin use on the blood loss outcome (Table 3, Fig. 4). Total dosage of oxytocin varied among the sample from 0 Units (no oxytocin used) up to 36.9 Units throughout the course of labor. Compared to G/G participants, A-carriers experienced 347.19 mL higher total blood loss (95% CI 174.25–520.13 mL) when oxytocin was *not* used during labor. However, when oxytocin was used in labor, for every one unit increase in total dosage, the *A*-carriers experienced 39.7 mL lower blood loss (95% CI, -69.48—-4.26 mL). In contrast, G/G individuals had 36.9 mL higher blood loss for every one unit increase in cumulative intrapartum oxytocin exposure (95% CI 10.7–65.7). Within the adjusted multivariable models, the main effects and the interaction terms remained significant with similar point estimates and only genotype and greater oxytocin dosage were related to blood loss. In these models, neither self-reported ancestry, primiparity, nor presence of genital tract lacerations were statistically associated with total blood loss.

**Table 3:** Interaction model of total volume postpartum blood loss by *OXTR* rs53576 and intrapartum oxytocin dosage, using a gamma distribution

	Model 1: Unadjusted β(95%CI)	Model 2: Adjusted β(95% CI)	Model 3: Adjusted β(95%CI)	Model 4: Adjusted β(95%CI)
**Main effects**				
AG/AA without oxytocin use	347.19 (174.25 – 520.13)***	390.07 mL (216.49 – 563.65)***	373.54 mL (193.02 – 554.07)***	371.39 mL (196.61 – 546.18)***
GG with intrapartum oxytocin (per Unit)	39.70 (11.72 – 67.68)**	41.22 mL (14.19 – 68.25)**	38.51 mL (11.31 – 65.71)**	38.17 mL (10.77 – 65.56)**
**Interaction**				
*OXTR *AG/AA (vs. *GG*) x per oxytocin Unit	-36.87 (-69.48 – 4.26)*	-40.63 mL (-72.30 – -8.96)*	-39.86 mL (-71.71 – -8.02)*	-40.56 mL (-72.35 – -8.56)*

Model 2: adjusted for self-reported European ancestry

Model 3: adjusted for self-reported European ancestry, primiparity

Model 4: adjusted for self-reported European ancestry, primiparity, genital lacerations

^*^ < 0.05, ** < 0.01, *** < 0.001

Following in the same pattern, A-carriers had a higher relative risk for additional pharmaceutical therapies to treat bleeding, atony and PPH. In the adjusted model, A-carriers had a 79% higher risk for needing at least one second-line uterotonic treatment (RR = 1.79, 95% CI = 1.08–2.95). This relationship remained after controlling for dose of intrapartum oxytocin, primiparity, ancestry, genital lacerations and active management of third stage labor. Adjusted interaction models between genotype and oxytocin dosage intrapartum also followed with main effects showing that G/G individuals had 6% greater RR for second line treatment with each increasing Unit of oxytocin total dosage, whilst A-carriers had 132% greater RR when oxytocin was not used during labor compared to G/G (RR = 2.32, 95% CI 1.16–4.67). The interaction term showed a trend toward a protective effect against need for additional treatment with intrapartum oxytocin use for A/G and A/A individuals (RR 0.96, 95% CI 0.92–1.002, *p* = 0.06).

## Discussion

The purpose of this study was to test the association of the rs53576 *OXTR* genotype with outcomes related to bleeding due to uterine atony following vaginal birth. In line with our hypothesis, we found that A-carriers were significantly more likely to have higher total blood loss than G/G homozygous individuals, with particularly high bleeding among homozygous A/A participants. Another key finding is that bleeding for A-carriers was also more pronounced among those for whom oxytocin was not needed/ administered during labor. Furthermore, we reported interactions between genotype and oxytocin dosage during labor, whereby G/G homozygous individuals had more blood loss with greater cumulative oxytocin use whereas A-carriers had less bleeding with greater intrapartum oxytocin exposure. Finally, relative risk for needing additional treatment, beyond the first line oxytocin therapies was also higher for A-carriers. To our knowledge, this is the first study to report an *OXTR* genetic variant is associated with postpartum uterine function and oxytocin response.

Lower blood loss and lower risk for PPH treatment for G/G individuals is concordant with what other researchers have reported in studies of rs53576 and oxytocin response when administered intranasally. The data from our study indicate the A-carrier participants appears to be less likely to respond to either active management of third stage labor (prophylactic oxytocin) or to first line treatment with oxytocin and thus required different classes of medications. In obstetric care, the first line treatment for heavy bleeding or PPH (despite any prophylactic oxytocin administration) is another, usually higher dose of oxytocin administered intravenously and/or intramuscularly. If this additional oxytocin is not quickly effective, then a provider will opt to add another uterotonic medication or opt for tranexamic acid to stabilize the fibrin matrix (preventing plasmin from binding and prevents the breakdown of fibrin clots at the placental attachment site). In the course of minutes, during an imminent hemorrhage, providers need to make decisions about which medications to administer, the sequence of which has been the source of much study [3, 55]. While onset of action is a primary consideration (oxytocin being among the fastest), knowledge of a person’s sensitivity to the medication could also be valuable in providing personalized treatment.

Overall, the molecular significance of the rs53576 variant has not been thoroughly described, however it has been related to differential gene expression [56], differences in OXT/OXTR levels in blood [57], and DNA methylation of *OXTR* [58] Few studies have examined oxytocin response or parturition related outcomes in relationship to *OXTR* genetic variability. In one study, researchers tested strips of myometrial tissue from 60 participants who underwent elective Cesarean birth, measuring contractility after oxytocin treatment in association with several *OXTR* variants [59]. However, in this study, spontaneous and oxytocin stimulated contractility between the rs53576 A-carriers vs. G/G groups did not differ over the course of 30 min/treatment. Furthermore, none of the variants tested were associated with *OXTR* mRNA expression in the uterine samples. A/A homozygotes for rs53576 were not analyzed separately and these participants had not undergone labor, nor was the SNP associated with other clinical outcomes; which limits the comparison of these associations relative to our findings. In a different study, researchers found G/G individuals had a slower rate of cervical dilation in labor, interestingly, this study reported the A/A frequency at 20% of the sample [60]. Another study of 151 pregnant participants [57] reported that A/A individuals (rs53576) had lower levels of oxytocin in serum as well as lower levels of OXTR on white blood cells among late-term gestation (41.2 weeks) compared to those giving birth at term gestation (39.3 weeks). This study did not compare other outcomes relevant to parturition, like oxytocin use. Our study, in contrast, did not find differences in gestational age by genotype, however, matching on labor induction between cases and controls could contribute to this balanced finding.

### Clinical implications

Predicting those at risk for PPH has proven to be challenging due to the multifactorial nature of PPH. As such, institutions and practitioners are charged with being highly vigilant for the possibility of PPH during all births [8]. While risk factors are well-documented from descriptive studies (i.e. longer second stage labor, infant macrosomia, oxytocin administration during labor); an estimated 40% of PPH occurs during births without these commonly-noted risk factors [8, 19, 61–64] as assessed with available screening tools. A reason for this inaccuracy in risk assessment is due to the fact that PPH is a diagnosis only made *after* a high cumulative blood loss has occurred. A person who experiences a PPH despite any prophylactic measures or PPH treatments performed is likely to be different in many ways from someone who was predisposed to have PPH yet had an adequate response to prophylactic or timely therapeutic measures and thus did not receive the diagnosis of hemorrhage. The definition of PPH (a diagnosis made after prophylaxis and treatment for heavy bleeding) therefore limits the understanding of the overall frequency of the *likelihood* of the problem occurring, thus does not quantify those truly *at risk*; instead it reflects those who experience PPH despite efforts to prevent/control bleeding [5]. Therefore, the accuracy of risk-prediction models (typically built off of electronic health record data samples) are highly influenced by provider-initiated practices designed to limit the occurrence of PPH in the first place. It remains plausible that people who hemorrhage despite the standard approach to prevention and treatment do so because they have other intrinsic features (e.g. genetic variants, epigenetic differences) that may influence the way that first line therapies (oxytocin) will work for PPH treatment. Precision methods in oxytocin sensitivity or PPH treatment, if proven, could help providers forecast a future hemorrhage or develop alternative strategies for individuals who are less responsive to first line therapies.

Our study highlights one subgroup in particular that may experience PPH without the commonly reported risk factors. While the AA homozygous individuals in our study were only 10% of the sample, 55.6% of the AA participants had a clinically defined PPH (> / = 1000 mL), accounting for 35.7% of all PPH. Furthermore, these individuals were concentrated in the distribution of the sample with very little or no oxytocin use during labor (Figs. 2 and 4). These individuals (as well as many AG individuals) seemed to progress adequately in labor, did not experience different genital trauma and their infants were similarly sized. Clinically, a PPH would have been less expected for this birth phenotype. However, if future studies replicate our findings that rs53576 can predict hemorrhage for people who otherwise score low-risk on current tools, it could be important in identifying a harder-to-predict group of people at risk for PPH.

### Research implications

Together, heterozygous A/G and homozygous A/A individuals also appeared to have higher blood loss cumulatively compared to G/G, though the significant interaction demonstrated that intrapartum oxytocin may have been more of a contributor to the postpartum blood loss for those with the G/G genotype. Future work on *OXTR* SNPS needs to consider how prior exposure to intrapartum oxytocin may mechanistically influence later oxytocin responses by genotype. For example, we would need to determine more clearly if specific genotypes affect primary myometrial receptor availability, oxytocin binding or other aspects of desensitization after oxytocin administration or intracellular signaling pathways in response to oxytocin binding.

Oxytocin exposure during labor has been reported to confer risk for PPH [5, 65, 66] (presumably via receptor desensitization and down-regulation [67])—however not all population-based clinical studies have reported this association [24]. This contradiction in reports, in light of our findings, may be a reflection of the mixture of genotypes that seem to respond differently to intrapartum oxytocin exposure and may influence the PPH likelihood. This variability in the response could be indicating the presence of an underlying pharmacogenetic oxytocin receptor sensitivity or vulnerability to receptor desensitization both of which could be tested in future work, both in vivo and in vitro.

### Strengths and limitations

The strengths of this study are in the recruitment of a clear phenotype for PPH cases, allowing for examination of a single etiology contributing to PPH (atony). We also gathered detailed oxytocin dose information and specific PPH management variables both pharmacologic and non-pharmacologic to help our clinical interpretation of the clinical presentation of PPH and the different classes of medications needed to control the bleeding. We also matched control participants on labor induction, this was done to not over-estimate the role of mode of labor onset in the association with PPH, which would inflate the oxytocin dose-to-blood loss relationship as well. Differences in location of birth may limit generalizability based on institutional or practitioner variations in practice. This study design, while the intended sample size was not fully realized, is still methodologically strong and contributes to the interpretation of the data linking atony-related PPH and genetic variation.

The focus on only one SNP could be viewed as a limitation in this study but also seen as a strength. The possibility that haplotypes within the *OXTR* or other genes in the oxytocin pathway may also be linked to oxytocin receptor function deserve further study, which may help improve prediction of oxytocin response or function. However, one valuable SNP uses fewer resources, and if replicable, could be sufficient to provide more informed care. This study should be replicated in larger samples to examine these associations within and between ancestral populations given reported differences in genotype frequencies.

## Conclusions

In sum, we provide evidence that a common *OXTR* variant is associated with greater risk for postpartum hemorrhage. Given that G/G participants had greater blood loss with higher oxytocin exposure, judicious oxytocin use during labor should also be considered as a way to help reduce PPH burden. A-carrier participants experienced greater postpartum blood loss and required more uterotonic treatments aside from oxytocin along with physical interventions (e.g. bimanual compression) to control bleeding. We found evidence of an interaction between oxytocin stimulation during labor and genotype on elevated or reduced blood loss after birth. We also highlight opportunities for advancing pharmacogenomic and precision-focused research for addressing this particular source of maternal morbidity during parturition.

## Supplementary Information

**Additional file 1**: **Supplemental Table 1**: Characteristics of cases of postpartum hemorrhage and controls among a sample of 95 vaginal births

## Data Availability

The datasets used and/or analyzed during the current study are available from the corresponding author on reasonable request.

## Abbreviations

PPH: Postpartum hemorrhage
OXTR: Oxytocin receptor
OXT: Oxytocin
SNP: Single nucleotide polymorphism
