# Supplementary material for: Oxytocin receptor single nucleotide polymorphism predicts atony-related postpartum hemorrhage
Source: BMC Pregnancy Childbirth. 2022 Nov 29;22:884. doi: 10.1186/s12884-022-05205-w (PMC9706912; doi:10.1186/s12884-022-05205-w)
Supplement: Supplementary file 1 — Additional file 1: Supplemental Table 1: Characteristics of cases of postpartum hemorrhage and controls among a sample of 95 vaginal births [file 12884_2022_5205_MOESM1_ESM.docx]

**Supplemental Table 1: Characteristics of cases of postpartum hemorrhage and controls among a sample of 95 vaginal births**

|  | Case (n=69) | Control (n=50) |
| --- | --- | --- |
| Demographic Characteristics |  |  |
| Maternal age (years), mean (SD) | 31.4 (4.9) | 32.4 (4.4) |
| Primiparous, n (%) | 40 (57.9) | 19 (38.0) * |
| Self-reported ancestry/ ethnicity  *(can have selected 1 or more)*  European ancestry, n (%)  Latin American ancestry/Hispanic, n (%)  Asian ancestry, n (%)  African ancestry, n (%)  Other, n (%) | 55 (79.7)  11 (15.9)  7 (10.1)  1 (1.4)  4 (5.8) | 39 (78.0)  1 (2.0) *  6 (12.0)  0 (0.0)  3 (6.0) |
| Location of birth, n (%)  Hospital  Community (home/birth center) | 68 (97.1)  2 (2.9) | 48 (96.0)  2 (4.0) |
| Antepartum Characteristics |  |  |
| Bleeding during first trimester pregnancy (self- reported) | 7 (10.0) | 1 (2.0) |
| Prior Cesarean birth, n (%) | 6 (8.8) | 4 (8.0) |
| Body Mass Index at delivery (kg/m^2^), mean (SD) | 31.9 (5.3) | 29.9 (4.7) * |
| Gestational age (weeks), mean (SD) | 39.7 (1.3) | 39.8 (1.1) |
| Third trimester hemoglobin (mg/dL), mean (SD) | 11.8 (1.2) | 11.9 (1.2) |
| Gestational diabetes, n (%) | 13 (18.8) | 5 (10.0) |
| Hypertensive disorder diagnosis, n (%) | 6 (8.7) | 4 (8.0) |
| Group B streptococcus (GBS) carrier, n (%) | 22 (31.9) | 8 (17.0) |
| Intrapartum Characteristics |  |  |
| Spontaneous onset labor, n (%) | 34 (49.3) | 26 (52.0) |
| Length of first stage labor (hours), median (IQR) | 15.1 (7.8-22.0) | 10.8 (6.2-16.7) |
| Length of second stage labor (hours), median (IQR) | 0.4 (0-0.9) | 0.3 (0.02-0.6) |
| Length of ruptured membranes (hours), median (IQR) | 6.1 (2.9-11.5) | 5.0 (1.2-8.6) |
| Infection/ chorioamnionitis suspected during labor, n (%) | 8 (11.6) | 3 (6.0) |
| Antibiotics administered (GBS + prophylaxis or suspected infection), n (%) | 30 (43.5) | 9 (18.8) ** |
| Epidural use in labor, n(%) | 49 (71.0) | 30 (61.2) |
| Oxytocin not used in labor, n (%)  Oxytocin used in labor, n (%) | 27 (39.1)  42 (60.9) | 22 (44.0)  28 (56.0) |
| Total oxytocin if administered intrapartum (Units), median (IQR) | 6.6 (2.0-16.8) | 2.2 (0.8-3.8) ** |
| Total oxytocin duration (hours), median (IQR) | 15.1 (8.1-24.0) | 8.5 (3.0-14.9) * |
| Highest maximum dose of oxytocin (mU/min), median (IQR) | 13.0 (8.0-18.0) | 8.0 (6.0-12.0) * |
| Instrument assisted vaginal birth, n (%) | 5 (7.2) | 1 (2.1) |
| Female infant, n (%) | 37 (53.6) | 21 (42.0) |
| Infant size (grams), mean (SD) | 3410.7 (568.5) | 3484.6 (689.9) |
| Postpartum Characteristics |  |  |
| Postpartum oxytocin used (Units), median (IQR) | 19.7 (10.0-25.4) | 10.0 (10.0-10.6) *** |
| Genital trauma (none/no repair needed), n (%) | 17 (24.6) | 14 (29.2) |
| Active management of third stage labor (prophylactic IM or IV oxytocin), n (%) | 59 (85.5) | 42 (84.0) |
| Timing of cord clamping <30 seconds, n (%) | 8 (12.5) | 3 (6.9) |
| Length of third stage of labor (minutes), mean (SD) | 10.0 (9.5) | 9.0 (10.4) |
| Total volume blood loss (mL), median (IQR) | 600.0 (500-900) | 250.0 (150-350) *** |
| Total volume blood loss (mL), range | 400-2540 | 50-600 |
| Difference in hemoglobin (admission to lowest postpartum value) (mg/dL), mean (SD) | -1.8 (1.8) | 0.05 (1.3) *** |
| Received iron infusion or blood transfusion, n (%) | 13 (18.8) | 1 (2.1) ** |
| Available DNA specimens, n (%) | 56 (81.2) | 39 (78.0) |

*p<0.05, **p<0.01, ***p<0.001
